# Sleep Disorders in Mild Cognitive Impairment

**DOI:** 10.7759/cureus.36202

**Published:** 2023-03-15

**Authors:** Bhawna Randhi, Sai Dheeraj Gutlapalli, Jingxiong Pu, Maheen F Zaidi, Maithily Patel, Lakshmi Malvika Atluri, Natalie A Gonzalez, Navya Sakhamuri, Sreekartthik Athiyaman, Pousette Hamid

**Affiliations:** 1 Medicine, California Institute of Behavioral Neurosciences & Psychology, Fairfield, USA; 2 Internal Medicine, Mayo Clinic, Rochester, USA; 3 Medicine, NRI Medical College, Chinakakani, IND; 4 Internal Medicine, California Institute of Behavioral Neurosciences & Psychology, Fairfield, USA; 5 Psychiatry and Behavioral Sciences, California Institute of Behavioral Neurosciences & Psychology, Fairfield, USA; 6 Research, California Institute of Behavioral Neurosciences & Psychology, Fairfield, USA; 7 Medical College, Aga Khan University Hospital, Karachi, PAK; 8 Family Medicine, California Institute of Behavioral Neurosciences & Psychology, Fairfield, USA; 9 Surgery, California Institute of Behavioral Neurosciences & Psychology, Fairfield, USA; 10 Surgery, Dr Pinnamaneni Siddhartha Institute of Medical Science, Gannavaram, IND; 11 Pediatrics, California Institute of Behavioral Neurosciences & Psychology, Fairfield, USA; 12 Pediatrics, Medical University of Graz, Graz, AUT; 13 Neurology, California Institute of Behavioral Neurosciences & Psychology, Fairfield, USA

**Keywords:** memory consolidation, sleep therapies, slow wave activity, alzheimer’s dementia, aging population, sleep disorders, cognitive impairment and dementia

## Abstract

We have an increasingly aging population and, therefore, cognitive impairment and dementia are becoming more common. Similarly, sleep disorders are also more common among the older population. There is a bidirectional relationship between mild cognitive impairment and sleep disorders. Additionally, both of these issues are underdiagnosed. By identifying and treating sleep disturbances early, we may delay the onset of dementia. Sleep helps in clearing metabolites like amyloid-beta (A-beta) lipoprotein. Clearance leads to decreased fatigue and proper functioning of the brain. A-beta lipoprotein and tau aggregates lead to neurodegeneration. Slow-wave sleep that decreases with aging is important for memory consolidation. In the initial stages of Alzheimer’s disease, A-beta lipoprotein and tau deposits were linked to lower slow-wave activity in non-rapid eye movement sleep. Improvement in sleep decreases oxidative stress which in turn leads to decreased A-beta lipoprotein accumulation.

## Introduction and background

An estimated 5.8 million people have dementia including Alzheimer’s disease (AD) in the United States alone. By 2060, this number is expected to increase to 14 million [1]. Total direct medical expenses due to AD-related dementias are expected to rise from $236 billion in 2016 to more than $1 trillion in 2050, according to the Alzheimer's Association [2]. Before progressing into AD, patients experience mild cognitive symptoms. Sleep is commonly affected in patients suffering from mild cognitive impairment (MCI). Sleep disturbances can include circadian rhythm disorders, slow-wave sleep (SWS) disorders, and others. Since it is difficult to identify people with MCI fairly early, they suffer from poor quality of life for a longer period.

Similarly, sleep disorders are also underdiagnosed; almost 70 million people in the United States have sleep disorders, but most of them are undetected in primary care centers [3]. There is increasing evidence that there is a bi-directional relationship between sleep and MCI. Sleep disorders start early in individuals with MCI as they suffer from frequent sleep disruptions. Also, cognitively healthy people who have a detectable amyloid beta (A-beta) in their cerebrospinal fluid (CSF) suffer from sleep disturbances [4]. Almost half of the patients with AD have some sort of sleep disturbance [5].

If sleep disturbance raises the risk of future AD, it's even more important to detect and treat those who have sleep disorders like obstructive sleep apnea [6]. Most of the management therapies we have for AD are mainly for symptom treatment. Given the increasing disease burden, there is a need for preventive therapies as well.

In the preclinical and early clinical stages of AD, sleep assessments could be utilized as markers of brain function, allowing for quicker and easier clinical trials of prospective treatments [6]. This article explores the pathophysiology of sleep disorders in MCI, if sleep disorders can be a presenting symptom of MCI, and if starting sleep medications prolongs the descent into dementia, especially alternative sleep therapies. Since benzodiazepines do not have a proper safety profile in the elderly [7], we can perhaps substitute them for safer alternatives like melatonin or light therapy [5].

## Review

Sleep is a vital part of our life, but it is often not given its due importance. Most of the time, we postpone sleep in lieu of other activities, go to bed at a later time, suffer the consequences the next day, vow to maintain better sleep hygiene, and then repeat this cycle. Sleep not only has many short-term effects but also long-term consequences. Arousal, alertness, attention, and prefrontal functioning appear to be hindered by sleep, possibly due to instability in the neurobiological systems in charge of attentional and sleep drives [8]. This review focuses on sleep, sleep disorders, and how this affects cognition. This literature review was done following the SANRA (a scale for the quality assessment of narrative review articles) guidelines.

How sleep affects AD pathophysiology

It is now well-known that sleep affects cognition. While awake, we make numerous decisions, which result in the formation and accumulation of metabolites. During sleep, there is an increase in the interstitial space in the brain, which leads to the clearance of metabolites from the brain like A-beta lipoprotein [5]. This clearance is done by the glymphatic system of the brain which is similar to the lymphatic system of the body, especially during the non-rapid eye movement (NREM) SWS cycle [3]. Clearance leads to decreased fatigue and proper functioning of the brain. In comparison to age-matched controls, insomnia patients with lower SWS had lower nightly declarative memory consolidation [4].

Reduced and fragmented SWS has been linked to greater levels of the amyloid-42 protein in the CSF in older individuals with normal cognitive function [9]. The pathological alterations that underpin AD are thought to begin 10-20 years before any cognitive symptoms occur, with the formation of amyloid plaques in the brain being the earliest detectable preclinical stage of AD [6].

**Figure 1:**
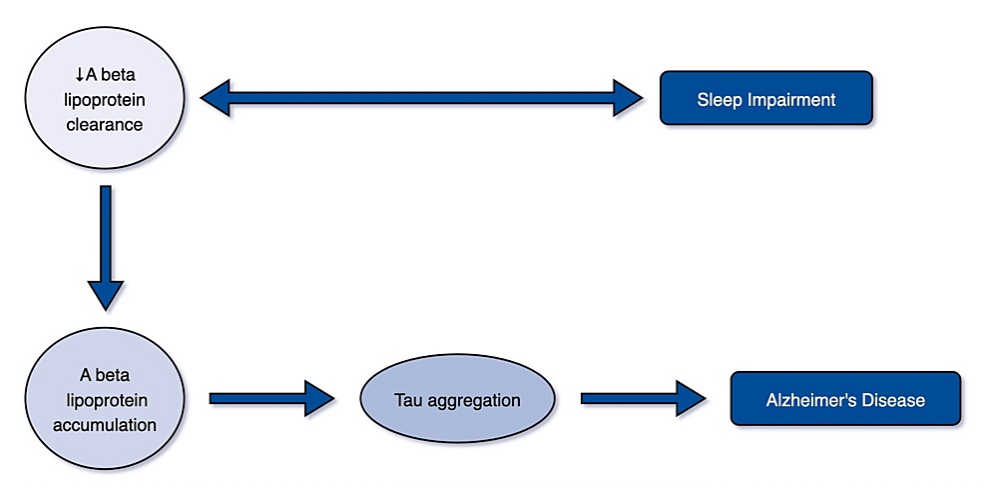
Role of sleep in the development of AD Author: Bhawna Randhi A-beta: Amyloid-beta

Toxic A-beta lipoprotein is hypothesized to start pathogenic events and lead to the creation of abnormal tau aggregates, which eventually lead to synapse loss and cell death, damaging neural circuitry [4]. Tau is a microtubule-associated protein found mostly in neuronal axons, where it helps to maintain microtubule (MIT) structure as well as synaptic structure and function. The tau protein is hyperphosphorylated in pathological states, and it dissociates from MITs, accumulating and producing neurofibrillary tangles [10]. Figure 1 portrays the role of sleep in the development of AD.

The brainstem has been identified to be the earliest brain region to acquire hyperphosphorylated tau, which may be linked to daytime wakefulness instability. Axonal transport is impaired by hyperphosphorylated tau and tangles [10].

The orexin (hypocretin) system is affected by A-beta lipoprotein and tau aggregation-mediated neurodegeneration. Orexin is responsible for maintaining alertness, and the lack of orexin is the primary cause of narcolepsy and cataplexy [10]. In studies in mice, both chronic sleep deprivation and orexin infusion raised interstitial fluid A-beta levels, whereas a dual orexin receptor antagonist reduced plaque development [11]. The number of orexin neurons in the hypothalamus is considerably reduced in AD patients in postmortem examination. The absence of or decreased orexin signaling appears to be linked to sleep issues in AD patients, especially in those who sleep excessively throughout the day [10].

Amyloid plaques lodge soluble A42, consequently, a decrease in CSF A42 (cerebrospinal fluid (beta)-amyloid 42 protein) indicates the existence of amyloid plaques. One study found that people with low CSF A-beta 42 had poor sleep quality. These people were cognitively intact; therefore, any amyloid accumulation would be regarded as a preclinical stage of AD [6].

Types of sleep disorders in MCI

Chronic insomnia has been linked to a slew of negative health outcomes, including a greater risk of high blood pressure, diabetes, obesity, depression, myocardial ischemia, and stroke, as well as a higher chance of cognitive decline and dementia [2]. General sleep alterations observed in healthy older people are increased nocturnal awakenings leading to fragmentation of sleep and decreased daily sleep duration due to equal reductions in both rapid eye movement (REM) and NREM. There is an increase in light sleep (NREM stages 1 and 2) and faster NREM/REM cycles [10]. When compared to age-matched controls, the proportion of SWS, duration of REM sleep, and sleep-wave activity (SWA) in SWS, EEG fall substantially faster in AD patients [10]. REM sleep has desynchronized EEG activity, while NREM sleep has synchronized activity. SWA stands for slow and delta oscillations, as well as isolated slow waves. The high quantities of SWA characterize Stage N3 (NREM Stage 3), also known as delta sleep or SWS. Slow oscillations are generated by the neocortex, and the thalamus helps with their stabilization by temporally altering cortical SWA [4].

One study found that changes in circadian activity rhythms were linked to an increased risk of MCI or dementia in 1,282 older women. Reduced amplitude, a less robust rhythm, and delayed peak activity timing were all implicated in the development of dementia or MCI [11].

A study done on women found that if there was greater variability in sleep efficiency, there was a 1.9-fold higher risk of developing MCI [12]. The above study also found that over the 4.9 years of follow-up, women with the poorest sleep efficiency had 1.5 greater odds of acquiring MCI or dementia, whereas women with longer sleep latencies had 1.4 greater odds of cognitive impairment [12]. Insomnia is characterized by trouble falling asleep, waking up during the night, waking up early and not being able to fall back asleep, and non-restorative sleep [2]. One study after controlling for other demographics/variables/factors showed that for each extra insomnia symptom, there was a 5% increased risk of incident MCI (HR = 1.05; 95% CI: 1.04-1.06) and dementia (HR = 1.05; 95% CI: 1.03-1.06) after 12 years of follow-up [2]. People with poor sleep efficiency spend a longer time in bed to make up for the lost sleep and get the same amount of total sleep time. A comprehensive study that used questionnaires to determine sleep duration indicated that an increase in self-reported sleep duration over time was linked to a two-fold greater risk of cognitive impairment [6]. Women who had a variable total sleep time had a 1.4-fold greater risk of developing MCI compared to women who had less variable total sleep time overall nights [12].

Excessive daytime sleepiness (EDS) can be brought on by issues with sleep hygiene, heart disease, obesity, drug use, depression, or sleep disorders [9]. EDS has previously been linked to diabetes, cardiovascular and cerebrovascular illness, depression, and other conditions that increase the risk of cognitive impairment. According to a study, even after accounting for all of these potentially confounding variables, there is still a substantial correlation between EDS and cognitive deterioration [13]. Participants with EDS were more likely to see a drop in MMSE scores (p = 0.006) [9]. EDS was found to significantly increase the probability of global cognitive decline by 30% while having no significant effect on verbal fluency or visual memory-related activities [13].

How sleep helps in memory consolidation

Memory consolidation occurs during SWS. By coordinating hippocampal sharp wave ripples with thalamic spindle activity during slow oscillation "up" states, cortical slow oscillations induce the reactivation of short-term hippocampal memories. This mechanism facilitates the consolidation of long-term memory in the neocortex by contributing to long-term synaptic plasticity alterations in neocortical networks [4]. Sleep-related memory consolidation was found to be reduced when SWS decreased with age [4]. According to a study, seniors with inconsistent rest-activity patterns fared worse on composite tests of executive functioning, memory, and speed than older persons with more regular rest-activity patterns [8]. That study also noted that older adults displayed worsening alertness, visual search, response times, word identification, addition, anagrams, and object use after sleep deprivation than younger adults [8]. In cognitively intact older people and the initial stages of AD, A-beta lipoprotein and tau deposits were linked to lower NREM SWA [4]. Researchers discovered evidence of higher atrophy in the CA2-4-DG region of the hippocampus in insomniacs compared to controls, which was linked to impairments in verbal and visual memory [2].

Interventions

Sleep disorders are common and almost always occur in conjunction with cognitive decline in patients. The Sleep Study Group of the Italian Dementia Research Association recommends they must always be thoroughly examined using an in-depth history, physical examination, and questionnaires and scales, directly written by the patient with the support of the direct caregiver, when possible [13]. Follow-up studies employing objective sleep/wake measurements, amyloid PET scan, and volumetric MRI assessment may be able to track changes in sleep patterns, brain amyloid burden, and neuronal damage in the elderly with cognitive impairment [9]. It is generally advisable to start with non-pharmacological means and then go toward pharmacological treatments. Some of the interventions are mentioned in Table 1.

**Table 1:** Pharmacological and non-pharmacological therapies TDCS, transcranial direct current stimulation; TMS, transcranial magnetic stimulation

INTERVENTIONS		
Non-pharmacological	Physical activity	
	Light therapy	Low light, bright light, ambient light
	Mechanical interventions	Rocking beds
	Noninvasive brain stimulation	TDCS, TMS
Pharmacological	Suvorexant, Donepezil	

Exposure to low light has been shown to improve cognitive function. Bright light therapy has also been found to help the elderly enhance and regulate their circadian activity rhythm and sleep. Furthermore, ambient light has been found to affect cognition and affective mood, and bright light possesses phase-shifting qualities that increase cognitive performance [11]. Acoustic stimuli have been proven to improve SWS, and this impacts memory recall in some patients [10].

Physical activity has also been demonstrated to have a favorable impact on circadian activity rhythms [11]. Rocking beds have been demonstrated to promote sleep by lowering sleep latency, boosting SWA, and decreasing nocturnal arousal. They've also been linked to better memory in people who are otherwise healthy [10].

Restoration of SWA sleep can be done by noninvasive brain stimulation techniques such as transcranial direct current stimulation (TDCS) and transcranial magnetic stimulation (TMS). In a study, TDCS was used to stimulate the brain at the slow oscillation frequency in MCI patients during a daytime nap; both slow oscillation power and memory performance increased. Moreover, repeated TDCS administrations during SWS caused slow oscillations and improved declarative memory retention the next day in both older and younger healthy people [4].

In one study, insomnia intervention included sleep deprivation, cognitive restructuring, good sleep hygiene, exposure to bright light, alterations in body temperature, and regular physical activity. Following treatment, the treatment group considerably outperformed the waitlist group in terms of sleep onset latency and sleep efficiency. In comparison to the waiting control, treatment was also linked to better performance on complicated vigilance tasks [8]. Improvement in sleep decreases oxidative stress which in turn leads to decreased A-beta lipoprotein accumulation [14].

The FDA approved suvorexant, an orexin receptor antagonist for treating insomnia in AD in the year 2020 [10]. For insomnia, it is recommended that pharmacological therapy be short-term and reviewed regularly every 4 weeks. Long-term usage of hypnotic medications should be considered only for specific indications [13].

Pharmaceutical therapies that target the gamma-aminobutyric acid system like benzodiazepine and zolpidem have been deemed to be less effective in treating sleep problems brought on by AD. It has been shown that they affect memory in both people and animals. Additionally, zolpidem use has been linked to an increased chance of dementia [10]. It has been demonstrated that barbiturates and benzodiazepines have detrimental cognitive consequences. Hence, when they are used on the elderly, it is possible that the MCI symptoms might not be due to neurodegenerative processes but rather these medications [14]. The risk of falls and fractures is also increased by long-term benzodiazepine use.

The cholinergic system, which encompasses memory and the sleep-wake cycle, has been linked to the malfunctioning of neurotransmitter systems involved in sleep. Cholinergic pathway neurodegeneration can also disrupt the ascending reticular activating system, resulting in daytime sleepiness and sleep disorders [5]. Donepezil, which is an acetylcholinesterase inhibitor, has been proven to increase the amount of REM sleep in AD patients [10]. Some people with MCI who are on cholinesterase inhibitors are frequently prescribed anticholinergics. Dual usage of these two drugs has been found to contribute to functional and cognitive impairment [14].

## Conclusions

This paper highlights the pathophysiology of sleep disorders in MCI, different types of sleep disorders, and different interventions that can be used to improve sleep, both pharmacological and non-pharmacological. Based on many articles, there is emerging evidence that sleep affects cognition. Sleep affects our visual and verbal memory, alertness, attention, and performance of simple and complicated tasks. Due to advances in medicine, people are living longer, and so the number of people suffering from cognitive impairment is also increasing. As having a good quality of life is fundamental, it is important to identify sleep disorders as early as possible and intervene. Therefore, there is a need to identify sleep disorders, especially in the elderly, and to investigate them and not just disregard them as a sign of aging. Information like how to maintain good sleep hygiene should be made available to the public. There should also be an awareness of the importance of sleep and its long-term consequences for the public.
